# Are Your Eyes “on the Road”? Findings from the 2019 National Study on Vision and Driving Safety in Spain

**DOI:** 10.3390/ijerph17093195

**Published:** 2020-05-04

**Authors:** Ignacio Lijarcio, Sergio A. Useche, Javier Llamazares, Luis Montoro

**Affiliations:** 1 Faculty of Psychology, University of Valencia, 46022 Valencia, Spain; lijarcio@uv.es (I.L.); luis.montoro@uv.es (L.M.); 2 Department of Technology, ESIC Business and Marketing School, 28223 Madrid, Spain; javier.llamazares@esic.edu; 3 Spanish Foundation for Road Safety (FESVIAL), 46022 Valencia, Spain

**Keywords:** vision, driving, visual health, Spanish drivers, road safety

## Abstract

*Background*: Vision is an undisputable contributor to the explanation of many human-factor related traffic crashes happening every day. The Inland Transport Committee (ITC), the United Nations regulatory platform, included on 1st April 2020 special action on the vision of road users inside the ITC Recommendations for Enhancing Road Safety Systems. The results of this wide-scale study on drivers’ vision health conducted in Spain perfectly illustrates the need of global action and its potential impact on the public health figures and the burden of potentially preventable traffic causalities. *Objective*: The aim of this study was to assess three key visual health issues (i.e., visual acuity, visual field campimetry and glare recovery) among Spanish drivers, in order to formulate implications and possible guidelines to enhance road safety. *Methods:* This cross-sectional study examined the visual health of a representative sample of 3249 drivers (70% females and 30% males) with a mean age of 41 (*SD* = 13) years, gathered from all the 17 autonomous communities of Spain. *Results*: The tests performed allowed to determine that 15% of Spanish drivers have a poor photopic vision, while 38% of them present an inadequate mesopic vision. Further, 23% of drivers have deficiencies in peripheric visual field campimetry, and the average time for full-vision recovery after a 10-s glare was 27 s. Sex, age and driver type (professional vs. non-professional) differences were found for the study variables. *Conclusions*: The findings of this study support the idea that certain demographic-based population groups of drivers present several unaddressed deficiencies and impairments in visual health. Overall an estimated 29.5% of Spanish drivers present visual issues, that need to be attended in order to enhance the prevention of driving crashes and the road safety of all road users.

## 1. Introduction

During the last ten years, the prevalence of visual impairments has systematically been growing in Spain [1,2]. This concerning trend has begun to represent a major concern for public health among public administrations, healthcare providers, researchers and health prevention and promotion practitioners, since visual health maintains a close relationship with population safety and welfare at all ages [3,4].

Visual defects and illnesses adversely impact the psychosocial and economic spheres of people’s life, but also the performance of different everyday activities, including operating machinery and motor vehicles [5,6]. Driving constitutes a considerably complex task, in which vision inarguably has a role [7], since it represents the most important information source on the road. Furthermore, numerous driving related injuries are associated with visual problems [8] and visually impaired drivers have more severe traffic crashes than healthy drivers [9]. In other words, an impaired vision can represent a major contributor in the likelihood of suffering a driving crash, as a consequence of not adequately receiving the needed incoming environmental information (*inputs*) that needs to be processed and responded to in a considerably short reaction time range, with potentially severe consequences in case of failure, as also emphasized at the *ITC Recommendations for Enhancing Road Safety Systems*, published by the United Nations Organization [10,11,12,13]. Further, applied studies have shown how drivers with certain eye conditions tend to decrease their exposure to the road and limit their driving to the safest times [14] as a manner of decreasing the risk of getting involved in a crash.

Also, recent social research outcomes point the fact that macro-social phenomena such as an aging population, that explains the increasing representation of elderly people into the driver’s census, may imply different new challenges for road safety practitioners, if factors such as the loss of visual acuity and other psychomotor impairments related to aging (inside and outside the visual sphere) are considered [10,15]. In the case of Spain, the aging of driving population has been catalogued as a latent threat to traffic safety. As a relevant figure, the official statistics reveal that for the year 2018, about 19% of the drivers were adults older than 65 but, at the same time, 27% of the deceased as a consequence of traffic crashes belonged to this age group. This fact, apart from illustrating the overrepresentation of older adults in traffic victim figures, raises different questions on how elderly drivers should be trained, assessed and monitored to strengthen their road safety [16].

In driving vision factors are more than merely age-related issues. Visual health has several other features, implications and risk groups than those directly explainable through age comparisons. In brief, visual impairments negatively affect the autonomy and functionality of subjects, being also related to poorer mental health, higher cognitive deterioration and greater probabilities of suffering accidental injuries [1,4].

### 1.1. Importance of Vision Testing for Driving Licensure 

In addition to knowledge tests on road rules, visual acuity testing is the most common functional method for determining a driver’s eligibility for licensure processes [12,17]. Indeed, most of the countries in which the psychophysical examination of drivers is mandatory have already included vision assessment as a necessary requirement for the qualification for a driver’s license (or for its renewal) [18]. However, these assessment processes only take into account certain basic visual parameters, and several other facets of visual function and processing, that are also involved in driving performance, are omitted in driver recognition exams [7]. For instance, and although visual acuity is crucial for safe driving (e.g., the reconnaissance of road patterns, traffic signals and other users’ behaviors), other relevant matters related to the driving task, such as campimetry and glare recovery, remain unstudied in driving recognition tests in many countries, although not in Spain. Accordingly, the use of visual acuity as a single criterion for assessing visual standards for driving has been widely criticized in the scientific literature [18]. Also, recent studies have related eye movements with attentional issues and fatigue, factors that may also explain a substantial part of the human-factor related traffic causalities occurring every day [19,20,21,22].

Worldwide, driver’s assessment is the only standardized process allowing the identification of “problematic” cases whose characteristics can put the road safety of all road users at risk, in order to take the required actions (e.g., specialized diagnosis, treatment, imposing driving restrictions) needed to reduce human factor-related jeopardies potentially deriving in economic losses, injuries and deaths on the road [23,24]. Driver assessment procedures are usually multidimensional, not being reduced to visual issues and the usually also address (although briefly) several issues related to the psychophysical health of subjects through the use of specialized tests, measures and protocols [25]. Nevertheless, some studies have criticized the laxity of some of the tests usually applied in these processes, including those employed for vision assessment, not strictly as a matter of rigor, as the examination is frequently reduced to visual acuity—necessary, but not sufficient—while other aspects may also play a crucial role in the accurate task of driving a motor vehicle [10].

Among several items and parameters that could be used for assessing visual health in driving, there are four issues that acquire special relevance for the accurate performance of this task and, according to the International Commission for Driver Testing (CIECA) [26], should be considered in driver recognizance and licensure processes:

First of all, visual acuity measures, both photopic and mesopic, have been frequently stated as relevant factors to consider for driver’ examination [27]. The photopic—high contrast visual acuity indicates the user’s visual quality in daylight conditions, and the mesopic visual acuity indicates the vision quality in low light conditions, such as at dusk or when there is fog or rain [28], providing additional insights in regard to functional vision loss processes that may constitute a risk under difficult driving conditions [29]. Secondly, driver’s visual field, measurable through pupil campimetry in response to perimetric stimuli [30], contributes to determine whether the subject has visual field defects in an efficient manner (through a quick machine-based screen testing), but needs to be contrasted with other indicators and at different angles (e.g., 45°, 60°, 80° and 90°) in response to the relative stability of the measures [31]. Thirdly, glare recovery constitutes another relevant factor to assess, given that, (especially during night driving) subjects need to face different light sources of high intensity or brightness [32], that may make difficult to detect road signs, patterns and other road users (such as the case of crossing pedestrians), prolonging the latency of reaction times [33].

Besides, much has been written in the literature in regard to the incidence of ametropia, which testing aims to determine the existence of refractive defects related to myopia and hypermetropia (or *hyperopia*), that may enhance the occurrence of driving errors (undeliberate risky road behaviors). Specifically, the prevalence of both myopia and hypermetropia has been determined as considerably high (but highly variant) in many populations, remarking the relevance of ethnic differences in this regard: myopia and hyperopia have an approximated prevalence to, respectively, 25% and 34% in South American countries [34]. In some Asian countries, approximately one out each four people suffer myopia (26.8%) and one out of six subjects (15.8%) presents hypermetropia [8]. Among Europeans, the prevalence of myopia reaches 30.6% (approximately one out of each three subjects), and hyperopia 25% (one out of each four subjects) of the population [35].

### 1.2. Vision and Driving Safety: Key Evidences and Gaps

As aforementioned, in the practical field visual health plays a crucial role for road safety. Several studies have systematically demonstrated how drivers with better visual parameters tend to perform less risky driving behaviors and to provoke less traffic crashes [9,11], contributing to improve one of the many facets of public health, taking into account that every year 1.3 million people die (and many others are injured) due to “accidents” that can be prevented through timely diagnoses and interventions [36].

However, and although several evidences remark the need of strengthening visual health as a way to face the threat of driving crashes, the panorama becomes certainly challenging when visual health statistics are faced. For instance, 65% of people who are visually impaired are aged 50 and older [37], bearing in mind that visual function, optical quality and intraocular scatter tend to change with age, creating optical deficits that need to be compensated in later stages of life [38,39].

Also, applied studies previously conducted in Spain have pointed out the existence of several gaps and inequalities in terms of visual health, between rural and urban regions, enhanced by factors such as access to information, eye care services and health promotion strategies (whose coverage can be substantially limited to urban areas) and, of course, as a result of economic issues [1]. These figures raise key questions in this regard, such as “to what extent are the driving population “healthy” in visual settings?”, and “may the eye care habits of drivers be strengthened via a higher healthcare coverage and further preventive strategies performed by institutional stakeholders?”.

### 1.3. Objectives of the Study

The purpose of this study was to assess three key visual health issues (i.e., visual acuity, visual field campimetry and glare recovery) among Spanish drivers, in order to determine their implications and formulate possible guidelines to enhance road safety.

## 2. Materials and Methods

### 2.1. Sample

The data used for this study was collected from a full sample of 3249 Spanish drivers (30% females and 70% males) aged between 16 and 90, with a mean age of *M* = 41.05 (*SD* = 12.98) years. The data was retrieved along the year 2019 across all the 17 regions (autonomous communities) of the Spanish territory. Nearly half (42.6%) of the drivers (39.5% of males and 49.4% of females) who participated in the study used some visual correction system. Within this group, 84.1% use glasses, 7.4% contact lenses and 8.5% both systems (glasses and contact lenses). Additional descriptive data of the sample are described in detail in Table 1.

An initial screening aimed to discard participants with severe visual pathologies that may impair the results of the study showed that 71.9% of drivers composing the final sample could be considered as emmetropes (no significant refractive defects were detected), and 28.1% of participants present slight or non-severe visual ametropic deficiencies (i.e., either myopia or hyperopia, a fact that remains undetermined as a consequence of the lack of specificity of the tool). Of the subjects with visual refractive defects 42.5% reported often suffering blurred vision symptoms, while 41.8% of them reported suffering blurred vision quite frequently. The rest of the sociodemographic variables (sex, age, etc.), and the type of driver (professional driver, driving days per week, etc.) did not show significant differences.

### 2.2. Study Design and Procedure

For this cross-sectional study, we used a non-probabilistic (convenience) sampling method, that is commonly used for this type of research focused on specific populations, founded on the accessibility to the population of interest (i.e., Spanish drivers) and on their willingness to partake in the study. Specifically, data collection was performed through a face-to-face invitation to drivers using the services of a national-covering network of gas and service stations (Spanish Petroleum Company—CEPSA, Madrid, Spain), present in all the regions of Spain.

All them were invited to answer to a questionnaire and to perform a standardized vision test (see Section 2.3 for further information). The global response rate (completed and totally answered questionnaires) was approximately 65%, from a total of approximately 5000 drivers initially asked to participate throughout the national territory.

The sample is highly representative from the national census of licensed Spanish drivers, that was composed of approximately 26,853,754 individuals for the year 2018 [40]. Sample size was initially calculated through the Raosoft^®^ sample size calculator (Raosoft Inc., Seattle, WA, USA), based on the total population and on the estimated sample needed to fulfill the basic parameters. The minimum sample size calculated was approximately 1843 subjects, assuming a confidence level of 99% and a maximum margin of error of 3%. The study sample was determined to be representative of the Spanish population considering not only its large size but also its concordance with the characteristics of the population and its geographical coverage. In this regard: (a) this study retrieved data from all the different 17 regions or autonomous communities (including two special jurisdictions outside the Iberic peninsula) of Spain; (b) a minimum quota, proportional to the population density of each one of the regions was established; and (c) subjects were invited to participate regardless their previous diagnosis in visual settings, avoiding selection bias for actively driving population.

As for procedural considerations, it is worth remarking that all the data were obtained from gas and service stations whose employees had been previously informed about the research project, and both the interviews and testing procedures were always performed by a trained member of the research team, especially considering the need of using advanced visual assessment tools, that need to be conducted by a professional in the field.

All the participants involved in the research were initially informed about the importance of answering honestly to all the questions raised in the interview, and to provide honest information for the performance of the vision test. Also important, participants were invited to enjoy a balanced snack of their choice, that could be picked up at the gas station store after completing the interview and visual test. However, the inexistence of potential monetary rewards as a consequence of their partaking in the study was explicitly emphasized before their participation.

### 2.3. Instruments

For this research, an assisted interview (conducted to complete a self-report-based questionnaire) and an applied vision test were performed. The average time required for answering to the questionnaire was approximately 10 min, and the vision test took approximately 5 min.

#### 2.3.1. Self-Reported Information

The interview was performed in Spanish and consisted of two main sections: The first part inquired about individual and demographic variables, such as age, sex, educational level, size of the town or city of residence, and occupation. It also contained a brief questionnaire about driving-related issues such as: crashes suffered while driving since they were licensed, driving tenure (years of experience as a licensed driver) and driving patterns/habits, that included: (a) the type of vehicle most frequently driven, (b) the approximate number of kilometers (km) driven every day, and (c) driving frequency (number of times they drive in a week), these last two measured with the objective of estimating mileage or driving intensity.

The second part of the interview was composed of a brief questionnaire aimed to assess attitudes and habits related to the vision in driving: perception of the risk of having poor vision and other risky scenarios for driving (ranging from 1 = not risky at all to 5 = very risky), self-perception of the quality of one’s own vision (ranging from 1 = very poor to 5 = very good), use of glasses or contact lenses, habits of vision checking, and any previously suffered vision-related problems.

#### 2.3.2. Visiosmart^®^ Vision Test

For the vision assessment, the Visiosmart^®^ 500 C87001 Automatic Vision Screener tool for vision assessment developed by Essilor Instruments (Chicago, IL, USA, 2018) was used. This is a standalone self-directed digital vision screener that can allow for automatic vision checks, which has been widely tested and certified according to ISO 8596 standard for this purpose. Visiosmart^®^ allows one to assess three key vision-related factors, that were strategically selected following the recommendations provided by the International Commission for Driver Testing [26]:

*Visual acuity*, i.e., a measure of the ability of the eye to distinguish shapes and the details of objects at a given distance [41]. Visual acuity is also one of the most used tests for driver licensing worldwide [42]. In this case, visual acuity was measured under two different lighting situations: (a) *Photopic acuity*—the user’s visual quality in daylight conditions, and (b) *Mesopic acuity*—the user’s visual quality under low-light conditions. The visual acuity values used in the Visiosmart^®^ test are represented on a continuous scale, in which the minimum possible value is 1 (very poor visual acuity) and the maximum 12 (maximum visual acuity) for both the photopic and mesopic acuity assessments. Based on this, the following guidance intervals are presented: [1,2,3] Very poor; [4,5,6,7] Poor; [8,9,10,11,12] Good.

*Visual field campimetry*, that objectively allows measuring the visual field by analyzing the pupil response to perimetric stimuli [30]. The Visiosmart^®^ test performs a repeated-measures assessment using different peripheral visual field amplitudes: 45°, 60°, 70°, 80°, 90° and 100°, in order to establish the cut-off point for each subject. For the interpretation of results, and in accordance to the specialized literature [18], 80° is suggested to be the cut-off criterion (adequate/inadequate), so that any subject who obtains a negative result at 80° or below, could be considered as with “deficiencies” in pupil campimetry, while those who do not have negative results or these are only at 80° or higher, can be considered as subjects presenting normal results in this test.

*Glare recovery time*, i.e., the extent it takes to a person to fully recover visual parameters and functionality after a certain time looking at a light, as it can also happen when a driver is glared when driving at night by another vehicle coming from the front with the high beams on. Thus, the temporary blinding effect from headlights is followed by a period of time required to fully recover vision, in which involuntary risky behaviors may take place. For this study, the criterion time of exposure to a light stimulus of 4 lux was established as 10 s, in accordance to the most common standards used in applied driving studies [43].

### 2.4. Statistical Analysis (Data Processing)

After a careful data curation, study variables were calculated. In the case of demographics, the data were rigorously coded and labelled, in order to categorize drivers by (e.g.,) sex, age and type of driver (professional/non-professional). The self-reported section of the interview (i.e., risk perception and importance attributed to vision in driving performance) were analyzed through descriptive statistics (mean, standard deviations, standardized errors). Each one of the four visual issues addressed by the Visiosmart^®^ test were scored and categorized according to the interpretation guidelines the tool. Additionally, mean comparisons (ANOVA and Brown-Forshyte’s robust tests for mean differences) were performed with the aim of assessing sex-based differences in the test sections providing continuous measures, for which confidence intervals [CI = 95%] were also calculated. As the sample was largely disproportional in regard to drivers’ sex (70.3% males and 29.7% females), the sample was weighted to a rebalanced distribution (54.2% male and 45.8% female drivers), more useful and adequate for carrying out sex-based comparisons without affecting means, standard deviations/errors, or confidence intervals of the contrasted variables. Frequency analyses were used to determine the age-based proportionality of sample segments (principally age groups). Bivariate (Pearson) correlations were used to examine the measures of association between visual parameters (when continuous) and demographic variables. All statistical analyses were performed using the Statistical Package for Social Sciences (SPSS), version 26.0 software (IBM, Armonk, NY, USA, 2019).

### 2.5. Ethics

This study was assessed and approved by the Ethics Committee of the Spanish Foundation for Road Safety (IRB HE2019003ESP), thus certifying that the research subject to analysis and its methods responded to the general ethical principles addressed in the Declaration of Helsinki for research with human subjects. This study did not perform any intervention and/or clinical trial on participants.

## 3. Results

### 3.1. Drivers’ Self-Rated Vision Sight and Road-Risk Perception

**Figure 1:**
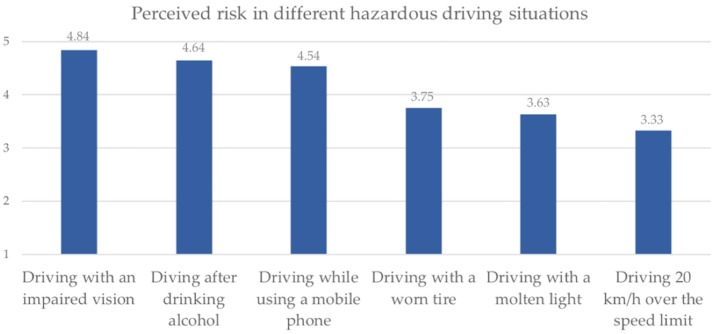
Average level of risk attributed to different potentially impairing driving situations (scale 1–5).

In order to assess the importance attributed to visual health in driving safety, drivers were asked to rate in a 0–5 scale (0 = no perceived risk at all; 5 = too much risk perceived) to what extent do they consider five different situations, that are generally conceivable as hazardous, may imply a risk for their driving performance, finding that “driving with an impaired vision” ranks on the first place with a mean of *M*= 4.84 (*SD* = 0.50), as shown in Figure 1, where females (*M* = 4.87, *SD =* 0.43) perceive a significantly higher risk perception on *driving with poor vision* (*F*_(13,247)_ = 12.532; *p* < 0.001) when compared to males (*M* = 4.82, *SD* = 0.52), but without significant differences between professional and non-professional drivers (*F*_(13,247)_ = 0.101; *p* = 0.751). Also, there was a significant bivariate correlation (*r* = 0.121; *p* < 0.001) between the age of drivers and the risk perceived in driving with an impaired vision; in other words, the higher is the age of the driver, the greater is the importance attributed to visual conditions for driving performance.

Drivers were also asked about their self-rated visual health. In this regard, 61.0% of them consider their vision is either very good (17.3%) or good (43.7%). 20.7% assess their vision as normal (not optimal but with no significant defects or impairments). Nevertheless, 12.9% of participants consider their vision as poor, and 5.4% as very poor. Non-significant sex-based differences (*F*_(13,247)_ = 0.003; *p* = 0.956) were found for self-rated vision between males and females, nor in comparisons between professional and non-professional drivers (*F*_(13,246)_ = 1.583; *p* = 0.208).

### 3.2. Visual Acuity

The categorical analysis of the obtained results indicated that 15.4% of Spanish drivers have a “poor” (15.1%) or “very poor” (0.3%) *photopic* visual acuity, even considering the optimal conditions of luminosity provided by the Visiosmart^®^ tool. The remaining 84.6% of drivers have an adequate visual acuity under daylight conditions. As for the low-luminosity condition, that is comparable to night driving, *mesopic* visual acuity was scored as “poor” (38.7%) or “very poor” (1.1%) for 39.4% of the participants of the study, while only 60.6% present acceptable values in this regard.

The average score for photopic visual acuity (under normal/high light conditions) was *M* = 9.95 for the full sample, and significantly different (*F*_(13,247)_ = 6.755, *p* < 0.001) when comparing between males (higher) and females (lower), as shown in Table 2. The mean found for the low light condition, i.e., mesopic visual acuity, was *M* = 7.82, without significant sex-based differences between male and female drivers.

Regarding types of driver (professional vs. non-professional), significant differences were found for both measures, being in both cases the means greater for the case of professional drivers: *photopic* visual acuity has shown significant differences with *M* = 10.32 (*SD* = 2.27) for professional and *M* = 9.85 (*SD* = 2.52) for non-professional drivers (*F*_(13,247)_ = 9.365; *p* < 0.001), and *mesopic* visual acuity has shown significant differences with *M* = 8.18 (*SD* = 2.52) for professional and *M* = 7.71 (*SD* = 2.53) for non-professional drivers (*F*_(13,247)_ = 9.150; *p* < 0.001).

**Figure 2:**
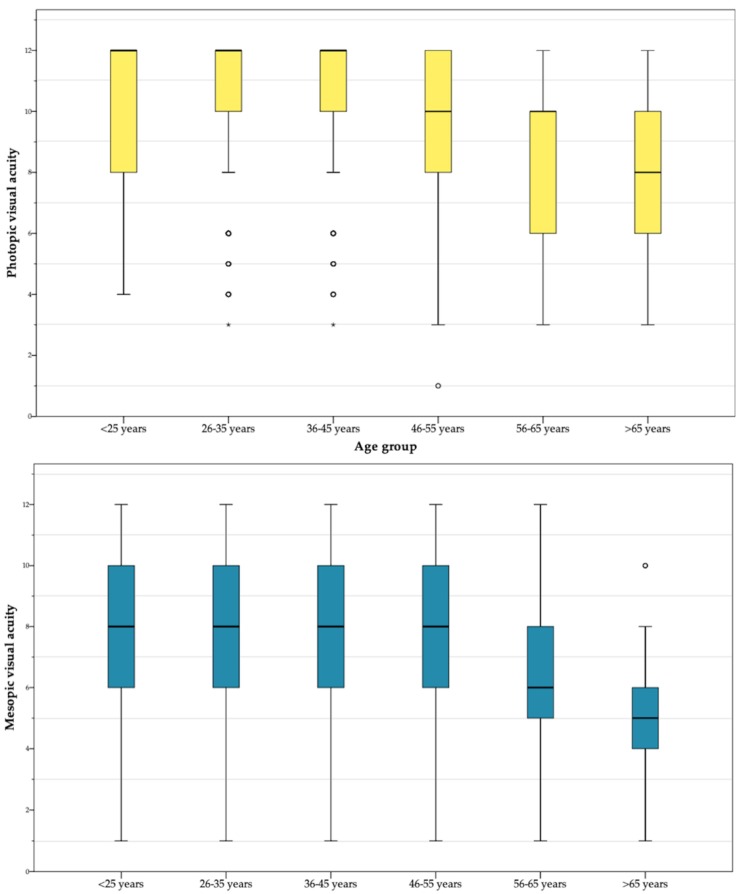
Boxplot-distribution of scores in photopic (high luminosity) and mesopic (low luminosity) visual acuity by age group (linear scale).

Furthermore, the age group-based analysis has shown a lessening trend for both visual acuity measures. The mean of *photopic* visual acuity decreases from the range *M* = [10.32–10.52] found for subjects younger than 45 years to *M* = 7.81 among individuals older than 65. Specifically, the Pearson correlation between age and photopic visual acuity was *r =* −0.216 ** (significant at the level 0.01). The mean values found for *mesopic* visual acuity -considerably lesser than the ones obtained by subjects in the photopic test- present also a decreasing trend with age, if the first three groups (ranging between *M* = [8.12–8.21]) are compared with adults over 65 (*M* = 5.25). Particularly, the Pearson correlation obtained between age and photopic visual acuity was *r =* −0.278 ** (significant at the level 0.01). Figure 2 displays the mean/dispersion-based distribution of the scores reported by both tests in subjects of different age groups.

### 3.3. Visual Field Campimetry

The results of the pupil campimetry tests are shown at Table 3, where the different openness levels evaluated from 45° to 100° appear, both for the left eye and for the right eye. For this test, 80° of field amplitude is established as a cut-off point, so that any driver who obtains a negative result at 80° or below could be considered as a driver as with “deficiencies” in terms of peripheral visual field campimetry, while those who do not they have negative results or these occur above 80°, it is considered as a driver that presents “*good/normal*” results in this regard.

Based on these criteria, the results of this study indicate that almost one in four drivers (23.5%) seems to have “deficiencies” in peripheric visual field campimetry. Further, 77.5% of males and 74.2% of females presented good/normal values in the campimetry test, with no significant sex-related differences found in this regard. Also, no significant differences as for the results obtained when measuring campimetry in the left and right eye were observed, being the percentage of right eye’s normal cases just slightly higher at critical values of 90° and 100°.

**Figure 3:**
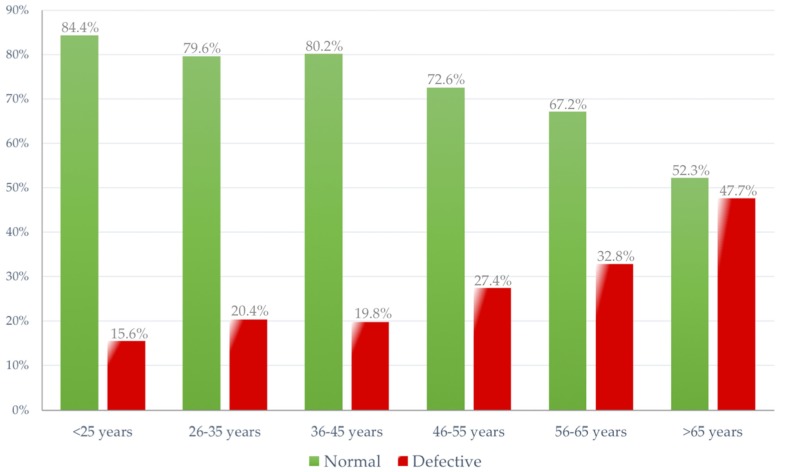
Distribution of normal and deficient cases for visual field screening (80° campimetry) by age group (percentages).

Drivers with good/normal results in campimetry are younger than those with poor results. The percentage of subjects with poor results increases especially after age 45, reaching 47.7% among drivers over 65 years. The average age of drivers with normal test results is *M* = 39.9 (approximately 40) years, while those with a defect in the campimetry test have an average age of *M* = 44.6 years. Figure 3 shows the graphical trends in terms of normal/defective cases across all age groups of drivers.

### 3.4. Glare Recovery Time

The glare test, as above indicated, measures the seconds it takes to a person to recover central vision after looking at a light for a certain amount of time. In this case, the criterion time for glare exposition was 10 s, i.e., the most common standard used worldwide for this type of test. The glare test has shown how, in 44.2% of cases, drivers need more than 20 s to fully recover normal vision parameters. Furthermore, of subjects 9.4% needed more than 60 s for full visual recovery, implying an excessive time driving under an impaired (partially functional) vision if a hypothetical driving scenario is considered. (Table 4)

Regarding the continuous measure (seconds) provided by the test, the average time required for full-visual recovery (considering all the sample) was *M* = 27.05 (*SD* = 26.86) seconds. In this regard, sex differences were also analyzed, finding marked significant differences between males and females. The average full-vision recovery time for males was *M* = 26.16 (*SD* = 27.22; IC_95%_ = [25.04–27.28]) seconds, while females needed *M* = 29.14 (*SD* = 25.89; IC_95%_ = [27.50–30.78]) seconds, and significantly different (*F*_(13,247)_ = 6.755, *p* < 0.001) when comparing the weighted samples. Summarizing the sex-based analysis outcomes, the glare recovery after a 10-s exposition takes in average 3 s more to female drivers. In other words, a male driver glared circulating at an assumed constant speed of 80 km/h may travel up to approximately 507 m. before fully recovering vision, and this figure could raise up to the 565 m. for the case of a female driver.

**Figure 4:**
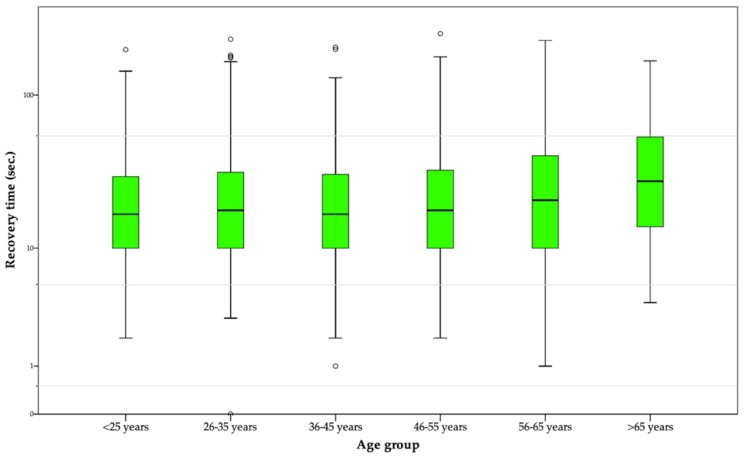
Boxplot—distribution of scores in full-vision recovery time after a 10-s exposition to glare, by age group (logarithmic/base-10 scale).

As for age-based analysis of glare recovery times, it ranged from *M* = 24.90 (*SD* = 24.06) seconds shown by drivers between 36–45 years [minimum] and the *M* = 38.39 (*SD* = 33.50) seconds needed to fully recover vision by drivers aged older than 65 [maximum], as shown in Figure 4 that graphically displays the distribution of recovery times in different age groups. Also, a significant Pearson’ correlation between driver’s age and recovery times (measured in seconds) was found, with r = 0.074 ** (significant at the level 0.01); in other words, the higher is the age of the driver, the greater is the visual recovery time needed after a glare. Going back to the previously described example, while full-vision recovery may take up to 482 m. to a driver ranged between 36–45 years, this travel distance can be increased up to 744 m. in an older driver.

Regarding comparisons between professional and non-professional groups of drivers, it was found that significant differences at the level *p* < 0.05 exist in the recovery time, being the *M* = 25.38 (*SD* = 25.22; IC_95%_ = [23.58–27.18]) seconds for professional drivers, and *M* = 27.55 (*SD* = 27.33; IC_95%_ = [26.48–28.63]) seconds for non-professional drivers (F_(13,243)_ = 4.158; *p* = 0.042).

## 4. Discussion

The purpose of this cross-sectional study was to assess three key issues (i.e., visual acuity, field campimetry and glare recovery tests) related to the visual health of the Spanish driving population, in order to assess its implications and formulate possible guidelines to enhance road safety.

The figures found in this national-coverage empirical experience, although showing an overall adequate set of visual parameters among the Spanish driving population, raise some concerning issues related to the visual health status and the road risk of subjects suffering visual conditions that may impair their driving performance.

A first positive aspect to highlight is the fact that, regardless their visual fitness, Spanish drivers (especially women) report a considerably high awareness on the relevance of visual health for both driving performance and road safety, that is a major concern for public health practitioners. In brief, the risk perceived in driving with visual impairments was greater than the risk attributed to other generic situations typically increasing crash risk, such as alcohol-impaired driving [44,45], problematic used of mobile phones in driving [46], and driving over the speed limits [47]. However, and although studies dealing with this and different other issues equally relevant to human-based crash prediction (such as fatigue, health and risky behavior) have endorsed the value of public awareness as a first step to take actions aimed at reducing road risks, further evidences support the idea that awareness in road safety issues is not sufficient to prevent drivers from having road traffic crashes, but foster actions, tools and focused interventions are needed to strengthen road safety figures [48,49].

In this regard, it is worth mentioning that, in vision settings, intra-subject awareness can be a matter whose reliability is arguable. For this study, 19% of Spanish drivers self-reported having any vision impairment, a figure that, when compared to the data retrieved from the three objective tests performed, raises several implications for strengthening driving safety through strategic actions aimed at improving the visual care and fitness of drivers.

Visual acuity tests under two different light conditions (first objective measure of this study) have not only shown that approximately 38% of Spanish drivers present considerable deficiencies in this regard, but that there are significant sex-related differences under normal light conditions. In other words, male drivers have a considerably better photopic vision than females, that is coherently with the results found by Shaqiri et al. [50] for a cohort of more than 600 adult participants. Furthermore, and according to the expected, age-group analyses show that both photopic and mesopic acuity are significantly lesser as the age of drivers increases, and older drivers are a primary focus of attention in this regard [42].

A similar result was found for visual field campimetry and glare recovery tests: in the first, age-based analyses made possible to establish, coherently to other studies in the field [51,52], an ascending slope in the percentage of defective cases that range between the 15.6% of deficient cases found in drivers aged under 25, up to the 47.7% of elderly drivers (over 65 years) having an insufficient peripheral visual field, in which no significant sex-based differences were observed.

As for the second, the recovery periods of male and female drivers were significantly different, indicating almost three additional seconds for the latter to recuperate full vision after being exposed to a light overstimulation, as might happen when driving in a two-way road and being glared by the headlights of another oncoming car that is circulating with the high beams on. Recent studies suggest that sex differences should be considered along with the driving patterns, such as the frequency of night driving and the type of trips performed across sexes; for instance, the percentage of males with poor vision that continue to drive at night is substantially greater than for female drivers [53]. In this regard, different studies have speculated about the higher incidence of different visual impairments among women, remarking that, given the biological origins of the great majority, they cannot be currently prevented but rather addressed through proper follow-up and treatment [50,54]. Given this gap, it would be interesting to wonder if something else, apart from the traditional restrictions (e.g., using glasses, not driving by night, that could be attended or not by drivers) might be done. For instance, and based on the previous fitness testing, periodic assessments among vision-impaired drivers could be performed with greater frequency, in order to gather accurate information on the evolution and potential treatment of these risk conditions, as happens with various pathologies in the case of the Spanish General Regulation of Drivers [55]. Although this potential follow-up is not a gendered strategy, it could contribute to protect the higher proportion of women presenting visual issues, since the current 10 years interval between obligatory assessments is a period that seems to be excessively long if a visual impairment is detected.

Furthermore and also related to specific groups of the sample, age differences seem to be one of the most generalized problem found in the three study variables addressed in this research, including the findings obtained for glare recovery tests, in which the time needed for full-vision recovery among older adults was almost 14 s greater than the time interval needed by young and adult drivers. This result is coherent to what was found by studies such as the performed by Schieber [56] and Gupta et al. [57], in which recovery times after a prolonged glare exposure were substantially longer for older adults, when compared to both young and middle-aged populations. In this regard, some previous sources have supported the idea that dynamic components of glare effects should be considered at the designing of different environments (including road scenarios) in which older population is involved [53,58,59].

However, it is worth stating that visual impairments are not exclusively an age-related matter. Evidences support that in the case of the most common visual illnesses among the adult population, including myopia and hyperopia, the highest prevalence is expectable between the second and third decades of life [34], being an early diagnosis and treatment crucial for preventing driving impairments or causalities attributable to visual lacks or deficiencies. Further, and as we pointed before, although key visual parameters seem to inherently fit better among young and middle-aged drivers, other potential factors such as fatigue, alcohol and drugs may substantially impair (temporarily or permanently) their visual fitness. For instance, previous studies have demonstrated how glare recovery times are considerably longer after risky behaviors such as alcohol consumption [57,60], that (although not exclusively) are more frequently observed in younger segments of the driving population [61].

### 4.1. Visual Health, Non-Professional driving and Driver Licensing: does the Current Model Need to be Re-Evaluated?

Another interesting finding of this study was that professional drivers tended to report better outcomes in visual acuity tests (both for photopic and mesopic conditions) than or non-professional drivers. This makes sense if it we bear in mind that, at least in most European countries, more regular check-ups of visual acuity are needed for a professional driver’s license, enhancing the early detection and treatment of potential impairments among high-intensity drivers [62,63], whose driving exposure and latent risk tend to be greater than in the case of non-professional drivers with less mileage.

This fact makes even more sense if it is considered that professional drivers are generally covered by regulated occupational health strategies and more systematic fitness testing, while non-professionals remain (outside the licensure and re-licensure context) conditioned to the performance of voluntary and extra-institutional visual revisions and controls, whose coverage is much less, if several social gaps in regard to the access to different healthcare resources are taken into account. However, the driver licensure system constitutes a universal process to what periodically all drivers (professionals or not) are subjected, and in which it is important to consider the need to carry out appropriate assessments that suitably diagnose the state of visual health of all road users [64]. As visual acuity, field campimetry and glare-recovery (tests that have shown good discrimination and interesting results in this study) are already assessed in most countries during driver’s fitness testing, the solution could pass through revising the frequency of these assessments. Thus, it is worth considering it as an ideal scenario for improving driving fitness through a more frequent revision of both visual and other relevant settings of physical fitness; whereas (as aforementioned) Spanish drivers need to perform medical fitness tests every 10 years (5 for those aged over 65), in some countries, elderly drivers must undergo a revision every year, strengthening the timely detection of potential impairments [65]. Also, previous studies have suggested the loss of driving privileges, i.e., the restriction to drive under certain conditions such as low luminosity, highways and high-risk paths, and the need of a more frequent assessment to monitor progressive eye diseases among visually impaired drivers [5]. Nevertheless, different barriers and gaps related to accessibility, information and economic issues in visual healthcare, expressed in (e.g.,) the lack of campaigns, public health strategies and interventions for at-risk population segments [66], remain pending to be solved.

### 4.2. Limitations of the Study

Although this study comprised the use of a large and representative sample of the Spanish driving population and advanced testing tools, there are some limitations to be acknowledged. Firstly, due to the convenience sampling method used, the sex distribution was dissimilar. However, sex-based comparisons were possible under optimum statistical parameters thanks to sample weighting, which is recommended when sub-sample groups substantially differ in proportion within a full sample. Secondly, this was not a clinical study; thus, and even though visual health professionals participated in our research staff and the basic settings (e.g., luminosity, noise, time to carry out tests) were controlled, we cannot make any clinical diagnosis based on the tests performed, since that would require several other tests and specialized procedures performed by professionals in individual assessment environments under conditions that cannot be replicated for our study population. Also, the number of vision-related study variables tested among participants was considerably limited and based in the advice of visual health professionals from an initial list of 15, considering the technical/logistic limitations that such massive data collection processes imply.

## 5. Conclusions

The results of this descriptive study support a relationship between visual health (assessed through four key skills) and driving fitness issues, including the risks perceived by subjects in the fact of driving with impaired vision.

Further, the existence of key age and sex-based differences suggests the need to target and address the higher-risk segments of the driving population, for both the performance of preventive and interventive actions on visual health, as well as in the revision of the current vision-related standards used for driving assessment and licensure.

This study endorses the value of healthcare practices for the development of behavioral skills to strengthen the prevention of human factor-related traffic crashes involving children and young people, under assumptions of well-designed programs, systematic interventions, continuous evaluation and improvement approaches.

### Practical Implications

Finally, three key practical applications of the study findings should also be discussed: Firstly, and although the panorama suggests attention to all drivers is necessary, sex comparisons highlighted a greater prevalence of visual issues among women. Concretely, female drivers have: (*i*) lesser visual acuity under daylight conditions than males and (*ii*) considerably longer glare recovery periods, that could compromise even more their driving safety, and actions are needed to reduce this gap. On the other hand, females have shown a greater risk perception while driving with impaired vision than males, a fact that could enhance their receptiveness to policies aimed at performing more regular vision assessments among “at visual-risk” drivers.

Secondly, age differences clearly point to drivers over 65 years as the most vulnerable group in regard to visual deficiencies, and particular attention must be shown on them, especially when their over-representation in traffic fatalities is considered [16]. Although in recent years elderly driver’s re-licensing criteria have been relatively improved, the European Commission still forecasts that, linked to the population ageing, for 2050 one out of three traffic fatalities will be an older person, which means an increase of 13% compared to the current rate [63]. Thus, more regular assessments could strengthen the proper detection and treatment of impaired visual conditions among elderly drivers.

Thirdly, more research efforts and practical actions aimed at improving the design of policies and strategic interventions in visual health and driving should consider not only the visual health-related attitudes and practices of the population, but the existence of key differences [66] in terms of visual health, information and accessibility to eye healthcare among age, region and income-based groups. Also, the authors would like to highlight the importance of developing inferential models for assessing the actual impact of visual impairments on road safety figures of drivers.

## Figures and Tables

**Table 1:** Characteristics of the sample used in the study.

Feature	Category	Frequency	Percentage
Sex	Male	2284	70.3%
	Female	965	29.7%
Age interval	≤25 years	409	12.6%
	26–35 years	775	23.9%
	36–45 years	902	27.8%
	46–55 years	717	22.1%
	56–65 years	310	9.5%
	>65 years	136	4.2%
Type of driver	Non-professional	2491	76.7%
	Professional	758	23.3%
Driving frequency	Once a week or less	70	2.2%
	2–3 days a week	251	7.8%
	4–6 days a week	807	24.9%
	7 days a week	2121	65.3%
Type of vehicle	Private car	2680	82.5%
	Motorcycle/moped/two-wheeled	171	5.3%
	Van/ Light Freight (>3.5 Tons)	200	6.2%
	Heavy Freight (<3.5 Tons)	18	0.6%
	Bus	11	1.6%
Have you been involved in a driving crash?	No	1808	55.6%
	Yes	1441	44.4%

**Table 2:** Results from visual acuity tests (photopic and mesopic) by age group, sex and overall sample.

Variable	Age Group	N	Total		Male		Female		Sex Differences
			*M* ^1^	*SD*	*M* ^1^	*SD*	*M* ^1^	*SD*	
Photopic visual acuity	<25 years	409	10.15	2.48	10.32	2.47	9.90	2.48	N/S
	26–35 years	775	10.34	2.33	10.40	2.28	10.22	2.42	N/S
	36–45 years	902	10.43	2.22	10.52	2.21	10.19	2.26	*
	46–55 years	717	9.68	2.53	9.78	2.54	9.05	2.49	N/S
	56–65 years	310	8.84	2.56	8.87	2.55	8.73	2.59	N/S
	>65 years	136	7.82	2.44	7.82	2.37	7.81	2.67	N/S
	Total	3249	9.95	2.48	10.01	2.47	9.81	2.49	*
Mesopic visual acuity	<25 years	409	8.26	2.61	8.34	2.66	8.14	2.55	N/S
	26–35 years	775	8.29	2.55	8.34	2.60	8.21	2.43	N/S
	36–45 years	902	8.37	2.48	8.46	2.50	8.12	2.39	N/S
	46–55 years	717	7.42	2.30	7.49	2.30	7.25	2.31	N/S
	56–65 years	310	6.35	2.07	6.37	2.05	6.30	2.13	N/S
	>65 years	136	5.46	1.76	5.53	1.70	5.25	1.97	N/S
	Total	3249	7.82	2.54	7.85	2.56	7.74	2.49	N/S

Notes: ^1^ Values ranging from 1 to 12; *SD* = Standard Deviation; N/S = mean difference is non-significant; * = mean difference is significant with *p* < 0.05.

**Table 3:** Results of peripheral campimetry tests under different field amplitude angles [45° to 100°].

Left Eye	Percentage of Cases	Right Eye	Percentage of Cases
*45°*		*45°*	
Normal	89.1%	Normal	89.9%
Defective	10.9%	Defective	10.1%
*60°*		*60°*	
Normal	95.7%	Normal	96.3%
Defective	4.3%	Defective	3.7%
*70°*		*70°*	
Normal	95.5%	Normal	95.7%
Defective	4.5%	Defective	4.3%
*80°*		*80°*	
Normal	94.7%	Normal	93.5%
Defective	5.3%	Defective	6.5%
*90°*		*90°*	
Normal	81.8%	Normal	82.2%
Defective	18.2%	Defective	17.8%
*100°*		*100°*	
Normal	44.5%	Normal	44.9%
Defective	55.5%	Defective	55.1%

**Table 4:** Time required for full-vision recovery after glare exposition (total sample).

Recovery Time	Percentage	Accumulated Percentage
<20 s	55.8%	55.8%
21–30 s	16.1%	71.9%
31–40 s	9.4%	81.3%
41–50 s	5.4%	86.7%
51–60 s	4.0%	90.7%
>60 s	9.3%	100%
